# Practical Lessons in Nursing

**Published:** 1887-07

**Authors:** 


### Reviews.

---

**Practical Lessons in Nursing**. The Nursing and Care of the Nervous and the Insane. By CHARLES K. MILLS, M.D., Professor of Diseases of the Mind and Nervous System in the Philadelphia Polyclinic and College for Graduates in Medicine; Neurologist to the Philadelphia Hospital; Consulting Physician to the Insane Department of the Philadelphia Hospital; Lecturer on Mental Diseases in the University of Pennsylvania, etc. Philadelphia: J. B. Lippincott Company. London: 10 Henrietta street, Covent Garden. 1887.

This book is the only one which treats of the care of the nervous. The first chapter is devoted to the care of hysterical and epileptic, as well as containing many useful hints on care of patients afflicted with chronic organic nervous diseases, sleeplessness, delirium, etc. Chapter second contains many useful hints on massage, movements and bathing. The other chapters are full of good suggestions in this much-needed field. It is well worth twice its price to any physician.

